# Neurophysiological Monitoring during Surgery on the Central Nervous System: The Role of Evoked Responses

**DOI:** 10.19070/2332-2780-1500031

**Published:** 2015-07-13

**Authors:** S Soghomonyan, GS Sandhu, N Stoicea, LN Kurnutala, S Cotterman-Hart, FL Christofi, SD Bergese

**Affiliations:** 1 Department of Anesthesiology, Wexner Medical Center, Ohio State University, Columbus, OH, USA.; 2 Department of Anesthesiology, University of Mississippi Medical Center, Jackson, Mississippi, USA.; 3 Department of Neurological Surgery, Wexner Medical Center, Ohio State University, Columbus, OH, USA.; 4 Department of Neurology, Wexner Medical Center, Ohio State University, Columbus, OH, USA.

**Keywords:** Neurophysiological Monitoring, Evoked Potentials, Electrooculography, Rhomboid Fossa Mapping, Cranial Nerve Stimulation

## Abstract

Neurosurgical, orthopedic and vascular interventions may be associated with an inherent risk of ischemia and structural damage to the central nervous system. Along with other modalities used to monitor the intraoperative nervous function, registration of evoked responses is intended to provide real-time feedback about the functional integrity of the central nervous system and help to prevent avoidable trauma during surgery. In this review, the principal indications and limitations of monitoring various evoked responses during surgery on the brain and spinal cord are discussed. Current approaches, recent advances and problems associated with intraoperative rhomboid fossa mapping, cranial nerve stimulation and electrooculographic monitoring are presented as well. The authors discuss the effects of general anesthesia on evoked responses and possible ways to avoid signal variability during registration. It is emphasized that only with close cooperation between neurosurgeons, anesthesiologists and neurophysiologists it will be possible to maximize the benefits of intraoperative monitoring of evoked responses and avoid misinterpretation of the results.

## Introduction

Real-time intraoperative assessment of central nervous structures, intends to decrease the perioperative morbidity and improve the outcome.

Several approaches have been used to monitor the intraoperative neurological function including patient awakening, and awake craniotomy under local anesthesia [1–5]. However, general anesthesia is required in majority of cases necessitating application of neuromonitoring. The intraoperative electroencephalography (EEG) is discussed elsewhere [6–9]. The current review focuses on intra-operative monitoring of evoked responses during interventions on the central nervous system (CNS).

## The Basics

Depending on proximity of the recording electrode to the signal generator, evoked potentials (EP) are categorized into near and far field potentials [10, 11]. EP are characterized by amplitude, polarity, absolute and interpeak latencies (IPL), and central conduction time (applicable to somatosensory EP (SSEP) [12–14].

Sensory EP (SEP) are generated by stimulation of sensory receptors, nerves or tracts, while motor evoked responses - by transcranial electrical, magnetic or direct stimulation of the motor cortex, corticospinal tracts, brainstem, cranial nerves (CN), nerve roots or spinal cord.

EP may occasionally be associated with false-positive or false-negative results [9, 15–17]. Clinical judgment and understanding the limitations of neuromonitoring modalities are required for correct interpretation of intraoperative EP changes. The optimal warning signal threshold level should be defined as choosing increasingly stringent warning criteria trades specificity for sensitivity.

The EP peak amplitudes are significantly smaller compared to EEG and require multiple stimulations, frequency filtering and signal averaging to be extracted from the underlying noise [12, 13]. Commonly, relative rather than absolute signal amplitude changes are used to assess intraoperative EP dynamics [13, 19, 20]. A latency increase (>10%) or a decrease in peak amplitude below a conventional threshold level (usually < 50%) are considered as significant and alert for potential risk of ischemia or structural damage [13, 19, 21]. However, these criteria are not based on empirical evidence, and are disputed by others.

Factors affecting intraoperative EP include ambient electrical noise, electrode type, impedance, placement site, their movement and dislodgement, equipment malfunction, preexisting neurological pathology, anesthesia, drugs, hypothermia, arterial hypotension, development of pneumocephalus, etc., [10, 15, 16, 21].

SSEP are used to monitor the integrity of ascending somatosensory pathways. The intensity, frequency and duration of stimulation depend on the nerve stimulated [12]. The evoked response is then measured along the somatosensory ascending pathway at Erb’s point (brachial plexus), C2–C7 vertebrae, or from the scalp over the primary somatosensory cortex [13]. A variation of this technique is the spinal cord-to-scalp stimulation [23].

SSEP are insensitive to motor impairment [15]. However, isolated intraoperative motor pathway ischemia and trauma rarely happen, and SSEP are being effectively used to monitor spinal function during surgery. Importantly, the lower extremity SSEP propagation includes additional components passing via spinocerebellar pathways, which may explain the method’s sensitivity to ischemia beyond the somatosensory zone (as cited in [11]).

Median nerve SSEP is not diagnostic for interventions below C8 [13], and tibial or peroneal nerve SSEP are used in such cases. Unlike upper extremity SSEP, stimulation of the posterior tibial nerve evokes a bilateral EP, probably due to the position of the somatosensory cortex in the longitudinal fissure [24].

**Figure 1.**
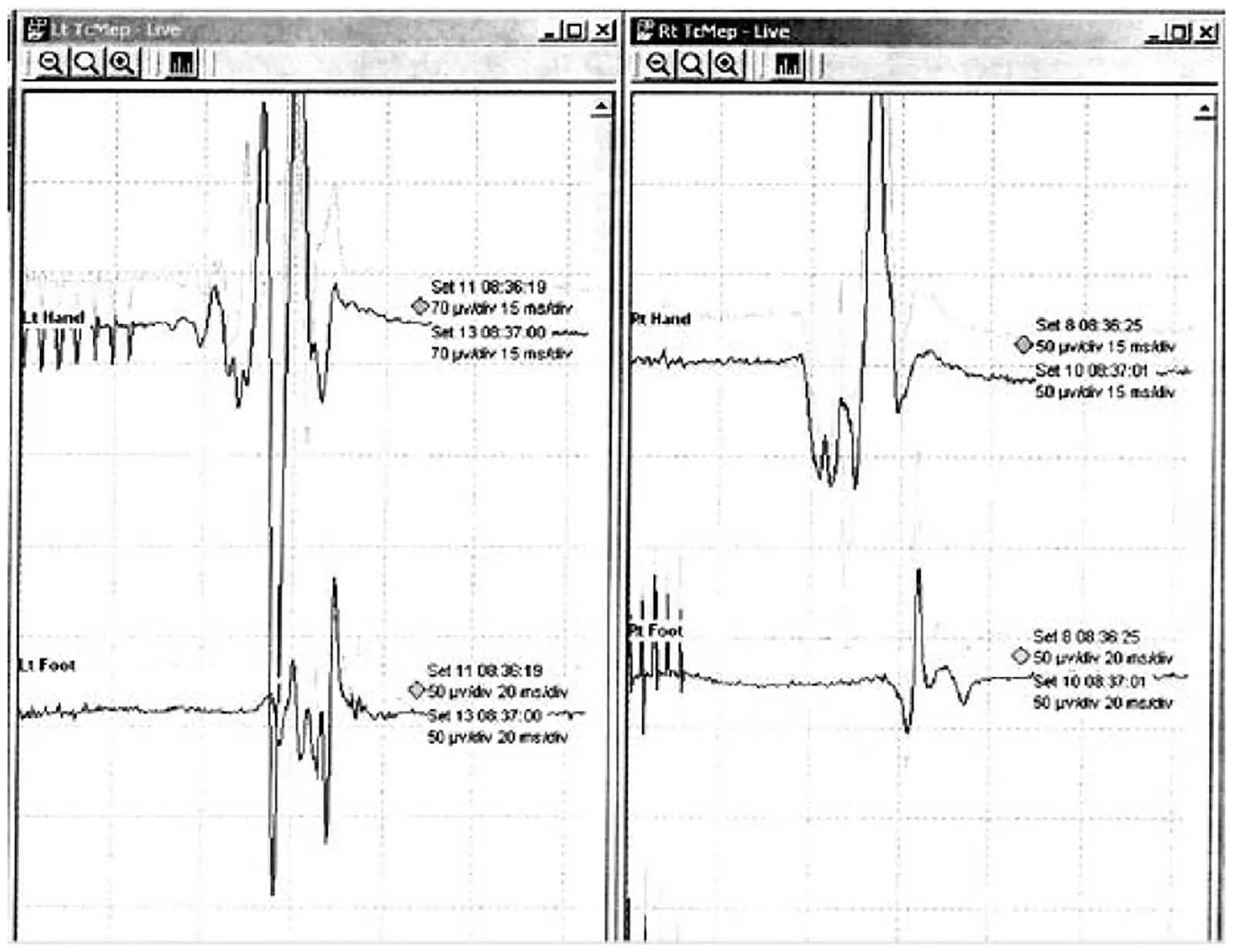
Normal transcranial motor evoked potentials (TcMEP). Anesthesia: desflurane combined with remifentanil, propofol and dexmedetomidine. The negative waves deflect up, and the positive signals deflect down.

**Figure 2.**
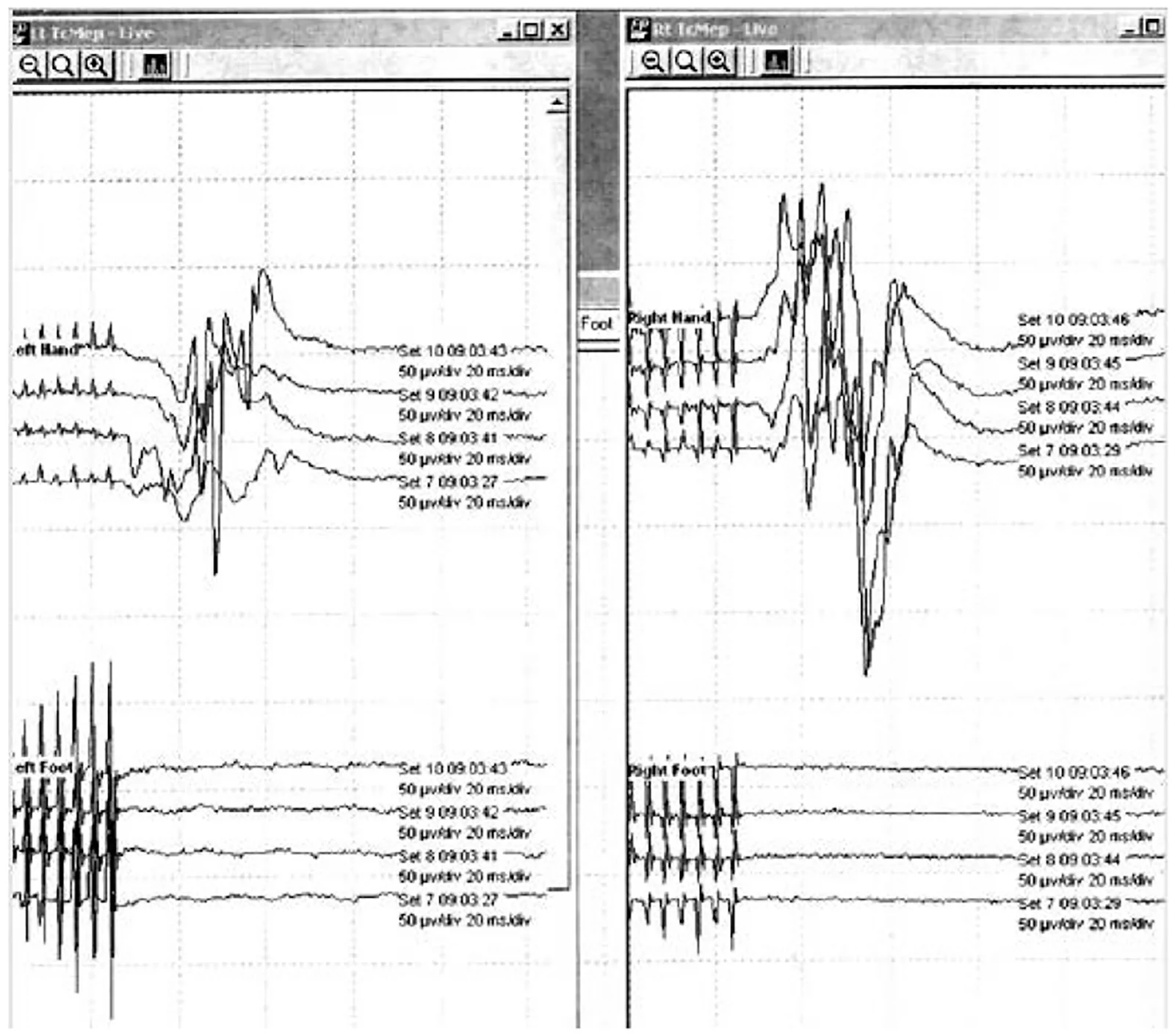
Absent lower extremity transcranial motor evoked potentials (TcMEP) in a patient diagnosed with spinal epidural abscess. Anesthesia: sevoflurane, remifentanil infusion. The negative waves deflect up, and the positive signals deflect down.

Motor evoked potentials (MEP) are used to monitor the descending motor pathways (Figure 1 and 2). Two commonly used methods of MEP registration are transcranial electrical motor evoked potentials (TcMEP) and transcranial magnetic stimulation (TMS). The latter method is mostly used for preoperative topographic assessment. Direct cortical stimulation may be applied during surgery within highly eloquent cortical regions [3] or during surgery on the brainstem and CN.

MEP is recorded at the spinal level (D- and I- waves) or from periphery - compound muscle action potential (CMAP) [13]. Smaller muscles of the hand or foot are recommended as sites for electromyography (EMG) and CMAP recording due to richer innervation. However, modern day multichannel recordings have the potential of detecting root-level injuries rather than just gross insults to the spinal cord. The MEP amplitude changes are more sensitive and specific than latencies [25].

Acoustic evoke potentials (AEP) are produced by applying click sound stimulation to the ears with subsequent recording of EP from vertex/mastoidal electrodes. A background “white” noise is applied to the contralateral ear to mask conductive noise stimulation. More commonly in the operating room, earphones with foam inserts are used to block external noise as well as minimize the crosstalk to the contralateral ear. Traditional AEP require a relatively long averaging period of approximately 90 seconds for each ear [9, 26]. Stable acoustic brainstem responses strongly correlate with preserved postoperative hearing, while their loss may not reliably predict the hearing outcome.

Brainstem auditory evoked potentials (BAEP) are commonly used to monitor the auditory pathways and brainstem integrity during surgery [10]. Most commonly, a 50% decrease in BAEP peak amplitudes and 0.5–1.0 msec increase in latencies on 2–3 consecutive trials are used as warning criteria during monitoring.

Electrocochleography (ECoG) and CN VIII compound nerve action potentials (CNAP) are used to monitor the acoustic nerve function during surgery. Their signals are significantly stronger compared to BAEP, and less averaging is required [13, 26].

Mid-latency auditory evoked potentials (MLAEP) are not applicable for intraoperative monitoring of auditory nerves because of high sensitivity to anesthetics. However, they may be used to measure the depth of anesthesia [11, 27, 28].

Visual evoked potentials (VEP) are monitored during procedures when visual pathways are at risk. However, this modality is not commonly used because of notorious variability under anesthesia. Intraoperatively, VEP are recorded from the vertex and occipital electrodes after a flash light stimulation to retina. The P100 component of VEP is associated with visual cortical activity. However, no exact neural generators can be linked to P100 because of polysynaptic nature of signal propagation.

## Effects of anesthesia on EP

Sensitivity of EP to anesthesia depends on the modality and pharmacological characteristics of anesthetic drugs. VEP are especially sensitive to general anesthesia due to the polysynaptic nature of signal transmission. SSEP are less sensitive, while the BEAP are relatively resistant to commonly used anesthetic doses.

A more detailed discussion of the interaction between general anesthesia and EP is out of the scope of this article and can be found elsewhere [10, 29, 30, 31].

EP suppression induced by inhalational anesthetics is more pronounced compared to intravenous drugs [10, 13]. Nevertheless, low doses of inhalational agents (0.5 MAC for TcMEP and up to 0.8 MAC for SSEP) can be effectively used during surgery combined with low-dose infusions of remifentanil (0.05 mcg/kg/min), propofol (50 mcg/kg/min), and/or dexmedetomidine (0.2–0.3 mcg/kg/h) [30, 33]. Their use will stabilize the anesthesia and reduce the risk of patient movements during critical stages of surgery.

Kempton and colleagues (2010) applied balanced anesthesia with isoflurane, N_2_O (in 94%), propofol (in 4.5%) and vecuronium for partial muscle relaxation in 247 patients undergoing scoliosis correction [34]. In 91%, the investigators recorded monitorable responses with anesthetic MAC levels > 0.5, while TcMEP could be recorded in 39% with MAC > 1.0.

Sevoflurane, owing to its solubility profile and fast elimination, is the induction agent of choice in young children, when EP monitoring is planned [35].

Most intravenous hypnotics suppress the EP in a dose-dependent way, while etomidate and ketamine increase SSEP amplitudes [10]. Opioid-induced EP suppression is proportional to lipophilicity and dose of the drug. In commonly used doses, opioids have minimal effects on EP and can be safely used during monitoring. High doses of remifentanil cause a 20%−80% decrease in P37 peak amplitude of SSEP with < 10% latency increase [36].

Benzodiazepines produce mild to moderate EP inhibition [10], which is less pronounced compared to inhalational agents. Administration of remifentanil and midazolam reduces the anesthetic requirements and increases the efficacy of EP registration [37]. Dexmedetomidine has been proposed as a useful adjunct to total intravenous anesthesia (TIVA) during monitoring [38]. It reduces the propofol requirements without affecting the anesthesia quality. The drug can be effectively combined with ketamine and fentanyl during intraoperative neuromonitoring (INM) [39].

Lidocaine (1.5 mg/kg/h) is an effective adjunct to general anesthesia during EP monitoring as it reduces anesthetic requirements and the incidence of patient movements in response to surgical stimulation [40].

MEP are more sensitive to inhalational anesthetics and relaxants, and therefore, anesthetic conditions optimized for TcMEP will usually produce acceptable SSEP [29, 41, 42]. Partial muscle relaxation can be used during MEP monitoring, which may even improve the signal quality, however, most of neurophysiologists refrain from using relaxants after tracheal intubation [13, 43, 44]. Direct cortical stimulation as well as increasing stimulation intensity and frequency can help to overcome some of the volatile anesthetic-induced MEP depression [29].

Another factor influencing the quality of EP monitoring is anesthesia stability. Bolus drug injections and changes in inhalational anesthetic dose may result in long-lasting EP suppression and should be avoided [29, 38, 41, 44, 45]. Besides choosing the right drug combination, it is important to keep the anesthesia depth and drug administration rate stable. When properly conducted, both inhalation and intravenous anesthesia generally yield anesthetic conditions appropriate for INM [41].

Intraoperative EP signal tends to degrade over time independent of anesthetic dose [46]. Signal fade is proportional to the anesthesia length and is more pronounced in young children and myelopathic patients [37, 46]. Differentiation between anesthesia-related signal fading and event-related EP degradation may be problematic.

Physiologic parameters should be maintained stable throughout surgery to exclude any interference with INM.

## Monitoring during spinal surgery

Spinal surgery carries the inherent risk of tissue damage with development of postoperative neurological deficit. INM during spinal interventions intends to reduce such risk by providing a real-time feedback to the surgeon [47]. Based on INM, modifications in anesthesia and surgery are made to avoid irreversible tissue damage [48].

**Figure 3.**
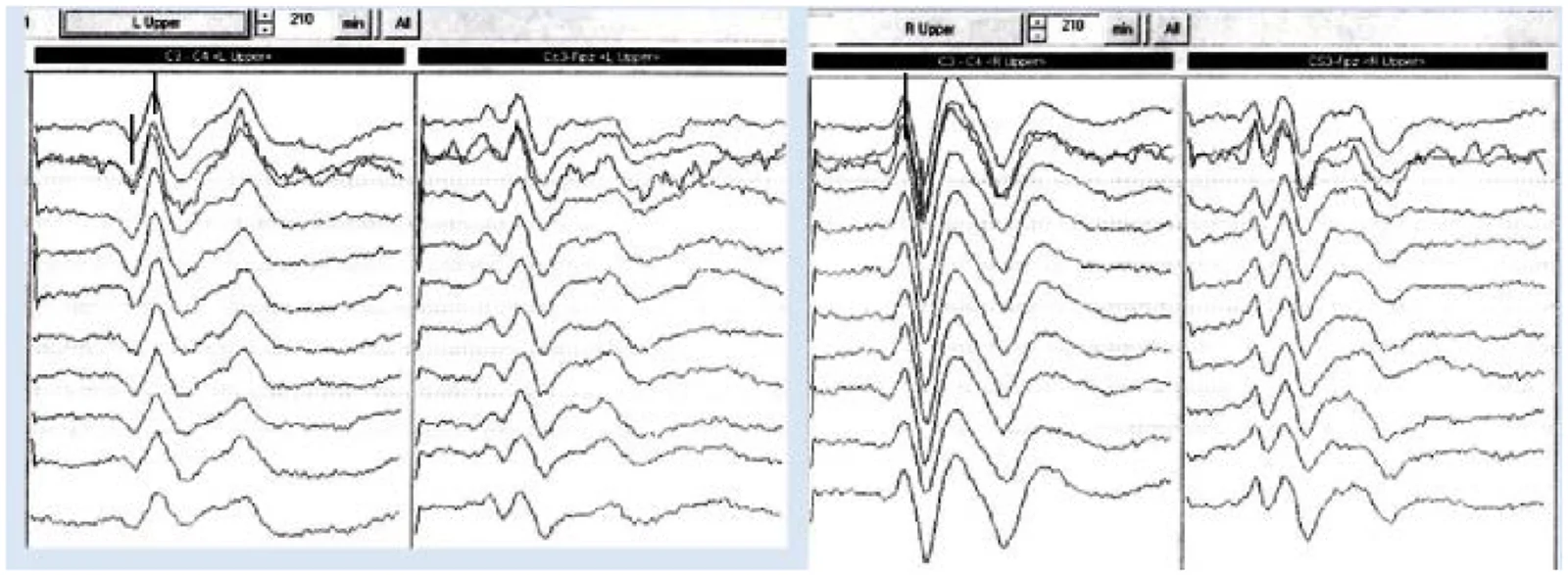
Somatosensory Evoked Potentials (SSEP) with stable right upper extremity responses and a decrement in the left upper extremity responses due to arm positioning (anesthesia: sevoflurane, remifentanil infusion). N20 (left mark), P23 (right mark).

Since their introduction, SSEP have become the most commonly used monitoring method in spinal surgery [12, 47]. Their main advantage over MEP is continuous registration throughout the anesthesia and surgery (Figure 3). SSEP do not preclude intra-operative use of muscle relaxants, which makes anesthesia management easier. Limitations of the method include sensitivity to anesthesia, hemodynamic changes and hypothermia [10, 49, 50].

The optimal stimulation intensity to induce SSEP is one that evokes a visible muscle twitch or a muscle twitch plus sensory threshold. It is determined by evoking a maximal amplitude nerve action potential recorded at Erb’s point – Fpz (median nerve) or popliteal fossa – medial condyle of tibia (tibial nerve) [51].

In young children, myelination is incomplete which may affect the EP [8, 12]. Neurodevelopmental disorders in children may present with higher EP thresholds, possibly, due to neuronal atrophy and altered synaptogenesis [52, 53]. Thus, neuromonitoring in pediatric patients requires experience of EP interpretation in this patient population.

Resection of the intramedullary spinal tumors is a high-risk procedure, and the most commonly used approach to such tumors is via dorsal median raphae. While electrical stimulation with subsequent recording of peripheral nerve or muscle responses is the standard approach, a newer technique with bipolar stimulation with subsequent SSEP recording has been suggested to identify the safe entry zone while approaching the tumor [54]. The safe incision zone is identified by characteristic phase-reversal of the EP. SSEP during upper cervical spine surgery may also detect brainstem ischemia caused by surgery [55].

SSEP and MEP have also been successfully used during syringomyelia surgery [56].

As a single monitoring modality, SSEP has a comparatively low sensitivity, and significant motor deficit may develop with preserved signal. On the other hand, intraoperative SSEP alerts may not be related with new neurological deficit in the postoperative period [57].

Combination of SSEP with MEP significantly improves the efficacy of monitoring [58]. Other fields of SSEP application include orthopedic, vascular surgery and procedures on peripheral nerves [59–64].

Spinal cord to scalp stimulation is an alternative although less commonly used method of SSEP monitoring [23].

A critical limitation of SSEP is the temporal summation, occasionally, requiring time intervals sufficient for permanent neurological damage [47]. Another inherent limitation is the low sensitivity in revealing isolated motor pathway injury [65]. Most of the studies reporting false-negative results with SSEP monitoring described insensibility of SSEP in cases of anterior spinal artery syndrome selectively affecting the antero-lateral column of spinal cord [23]. Any electrophysiological test will provide information only on specific neural structures and pathways propagating the signal, and there will always be clinical situations when local isolated damage to a region adjacent but functionally unrelated to the monitored pathway will remain unrecognized. Naturally, neither SSEP nor TcMEP are very good for predicting clinical deterioration in the opposite test’s neurological counterpart [66].

MEP require more restrictive anesthesia requirements, may cause patient movements and have less clearly defined criteria for raising the alarm [48]. Nevertheless, MEP became a significant step towards safer and less traumatic surgery.

Merton and Morton (1980) were the first to describe transcranial electrical stimulation in humans [67, 23, 47]. Later on, application of high-frequency multi-pulse electrical stimulation with relatively low voltages improved the reproducibility of the method and, along with improvements in anesthesia protocols, led to widespread use of MEP during spinal surgery [47].

MEP can be evoked via transcranial or direct cortical stimulation. However, the noninvasive transcranial stimulation is the preferred method [15].

Numerous methods to monitor the motor pathways have been suggested: spinal cord to spinal cord technique, neurogenic and myogenic MEP, triggered and free-running EMG, recording the D- and I-waves and CMAP, which all have their indications, advantages and limitations [8, 20, 23, 47]. Among them, the most commonly used methods are D-wave registration using epidural or spinal electrodes and CMAP recorded from the muscles of interest. These methods can be applied separately or in combination with free-running EMG.

D-waves reflect direct activation of the corticospinal pathways and are used mainly during surgery on proximal (above T_10_) spinal cord [68, 47, 15, 23, 69]. A 20–50% decrease in D-wave amplitude or a 10% increase in latency is indicative of possible postoperative neurological deterioration [47, 70–72].

EMG recording of transcranial MEP (CMAP) allows for assessment of the entire motor pathway including the peripheral nerve. [47] During monitoring, muscle relaxants generally should be avoided and anesthesia levels kept stable, although some centers prefer partial muscle relaxation during surgery.

For TcMEP monitoring, muscle relaxants are used for intubation only, and if they still are to be used, a target T_2_/T_baseline_ of 0.5 is recommended [43, 73].

Muramoto et al. (2012) suggest using absolute and relative (to baseline) cutoff CMAP amplitudes (12%, 1.9μV and 25%, 3.6μV, respectively) as indicators of neurological deterioration [18]. Threshold stimulation level (increase of threshold stimulus > 100 V) can be used as a criterion to detect intraoperative neurological deterioration ([66] as cited in [72]).

CMAP is more sensitive to ischemia than D-wave and SSEP, however, the D-wave amplitude correlates better with long-term motor outcome (as cited in [9]). A critical weakness of CMAP is the intermittent character of monitoring [47].

Adverse effects of TcMEP include cardio-vascular reactions, metabolic acidosis, tongue laceration, patient movements, and seizures [53]. TcMEP synchronization with ECG may help to avoid arrhythmias [63]. The possibility of false positive TcMEP should also be considered [74].

Triggered EMG is used to detect medial pedicle breach by a vertebral screw and is used in minimally invasive spinal surgery [15, 47, 72].

The main advantage of free-running EMG over the other methods of motor pathway monitoring is continuous data acquisition [47]. The method is not specific and it is sensitive to changes in anesthesia and temperature, pre existing neurological deficit, electrical artifacts, and electrode positioning [8]. EMG combined with intermittent TcMEP has been suggested as a better way to monitor the motor pathways [75].

H-reflexes and F-waves strongly depend on anesthesia level and are not currently considered part of a standard clinical practice [72].

## EP monitoring during surgery on the brain

### VEP (CN II)

The reported incidence of postoperative visual deterioration reaches 10% and varies from 3% to 38% among patients undergoing surgery for ophthalmic aneurysms, epilepsy and tumor resection in the vicinity of the optic tracts, chiasm and pituitary area [76, 77]. Postoperative visual loss has been described even after spinal surgery [58, 78, 79]. VEP monitoring is indicated during surgery in vicinity of the visual pathways and during neurovascular procedures [10, 77, 80]. After initial enthusiasm, intraoperative VEP recording failed to show consistency with no clear development over decades of standard protocols for this monitoring technique (as cited in [81]).

Many literature reports show lack of correlation between the VEP and postoperative visual outcome attributable to preexisting visual dysfunction, technical difficulties as well as changes in anesthesia and physiological parameters [10, 11, 76, 82, 83].

The reproducibility of VEP during transsphenoidal surgery is 89.6%, which is much lower than that for SSEP or BAEP [82].

Other reports indicate on usefulness of intraoperative VEP [9, 76, 80, 83, 87]. Goto et al. (2007) reported of a patient with paraclinoid aneurysm where the superior hypophyseal artery (SHA) originated from the aneurysmal body [88]. During surgery, the VEP reversibly disappeared every time after attempts of SHA temporary clipping. Surgery was modified, and a permanent clip was placed on the aneurysm body sparing the SHA. The patient had no visual disturbance postoperatively.

Application of specially designed goggles and discs for stimulation, co-registration of electroretinogram, use of TIVA and other modifications help to overcome the technical obstacles and improve the diagnostic value of VEP [80, 83]. Unfortunately, impaired preoperative vision is a major predictor of postoperative deterioration, and a strong dependence of VEP on intact vision constitutes a significant limitation [89].

Several authors used invasive methods of intraoperative visual evoked response registration with encouraging results [75, 84–87, 90–92].

Despite the controversies and conflicting results, recent advances in technology and anesthesia technique enhance the enthusiasm towards VEP and justify further research in this field.

### Olfactory EP (CN I)

Anterior cranial fossa surgery is related to an increased risk of loss of the olfactory function [77, 93, 94].

Anosmia may have a significant impact on quality of life, as depression and decrease in overall satisfaction with life have been associated with this complication [77]. Sato et al. (1996) proved experimentally and clinically the feasibility of intraoperative registration of stable and reproducible olfactory EP [94].

The method is not currently used in clinical practice, and requires further research.

### Rhomboid fossa mapping and CN monitoring

Rhomboid fossa surgery requires high precision and carries the risk of serious trauma to vital structures [8, 95–98]. The brainstem contains vital centers, nuclei and pathways concentrated within a small volume, making surgery in this area extremely challenging. The situation becomes even more complex with distortion of normal anatomy by the growing tumor.

SSEP and BAEP together monitor only 20% of the brainstem (as cited in [98]) and, therefore, monitoring the motor function is important to increase the surgical safety and better control the extent of tumor resection.

Both mono-and bipolar and either constant-current or constant-voltage stimulation can be applied to locate the CN and their nuclei before tumor resection [95, 96, 99]. Stimulation intensity commonly starts at 2.0 mA and is reduced to the threshold level once the motor nucleus or CN is identified, or it can start at low levels and then progressively increased to elicit CMAP from the monitored muscle [98–100]. Stimulation of CN IX–X can evoke hypotension and bradycardia, while current intensities exceeding 2 mA may trigger cardiovascular reactions (as cited in [98, 99]).

For security reasons, the stimulation of any point should not exceed 5 seconds [97]. Negative stimulation results must not be interpreted as absence of CN nuclei under the probe. Instead, repetitive stimulation may be required through the tumor mass during resection in order to detect the CN nuclei [96].

Rhomboid fossa mapping monitors only the efferent pathways of the brainstem reflexes, and the patients may still develop bulbar dysfunction despite uneventful stimulation.

Monitoring the lower CN (IX-XII) during resection of low brainstem lesions is important to avoid associated atrophy of tongue muscles, dysphagia and loss of the cough reflex [11, 17, 95].

Evoked responses or free-running EMG can be registered using specially designed wire electrodes with 2-mm bare hook tips inserted into the soft palate or pharyngeal wall (CN IX), false vocal cords (CN X), lateral wall of intrinsic tongue muscle (CN XII), and trapezius muscle, (CN XI) or, alternatively, surface electrodes attached to a laryngeal mask or endotracheal tube may be used [17, 95, 96, 98, 99, 101, 104].

Spontaneous EMG recorded from the false vocal cords can be used effectively to monitor the recurrent laryngeal nerve function during anterior cervical discectomy and fusion [105].

### Monitoring CN VII

CN VII is frequently monitored during cerebellopontine angle (CPA) and rhomboid fossa surgery [11, 95, 97]. To monitor the facial nerve, EMG, observation, video-monitoring, CMAP, and MEP can be used.

High frequency (>30 Hz), high-amplitude (100–200 μV) and long-lasting trains on facial EMG are related to worse postoperative facial nerve outcome [26].

An alternative or, perhaps, supplementary method to the intraoperative facial EMG registration is intraoperative direct observation or video-recording of the facial muscular activity [26].

**Figure 4.**
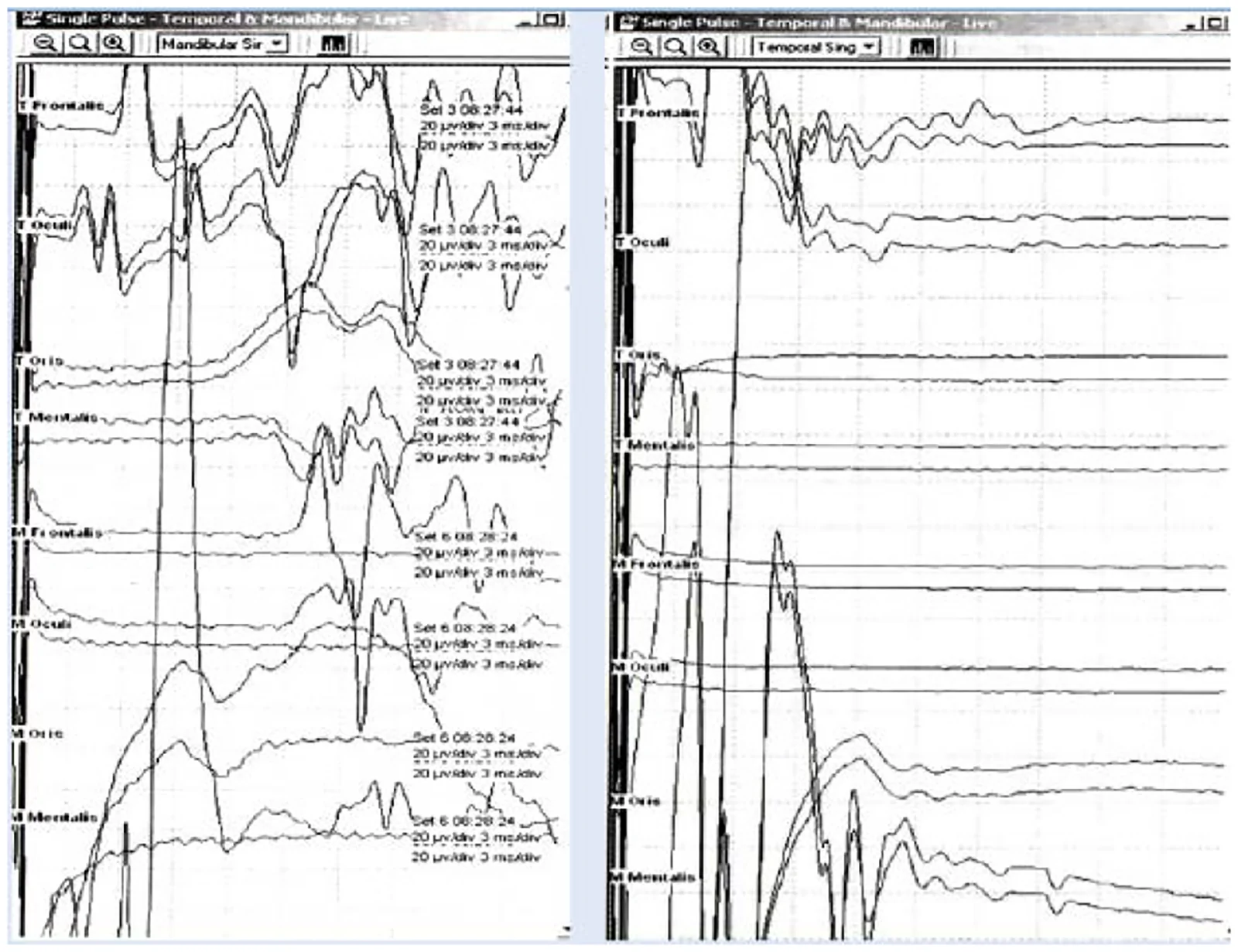
Electromyographic (EMG) responses to peripheral facial nerve stimulation with lateral spread before (A) and no lateral spread after (B) microvascular decompression in a patient undergoing surgery for hemifacial spasm on the left side (anesthesia: desflurane, remifentanil, propofol).

Registration of CMAP following electrical stimulation of CN VII or the brainstem is a method of intermittent monitoring of the nerve integrity. To provide a real-time feedback to the surgeon, the evoked EMG signal triggers a sound alarm [26]. It is important to remember that CMAP registered after the intracranial stimulation will depend on stimulation intensity and allow for monitoring only the section from the stimulation site to the recording point, even though normal functioning of facial muscles requires integrity of the whole pathway [106–108]. Another limitation is the possibility of signal spread after facial nerve stimulation (Figure 4).

Intraoperative monitoring of facial MEP allows for monitoring of the whole facial motor pathway [97]. A high correlation exists between the postoperative nerve function and final-to-baseline MEP ratio [106]. Initial contralateral single-pulse stimulation is recommended before recording the MEP to exclude any signal misinterpretation caused by direct electrical extracranial spread. [100, 106]

### Monitoring CN V

Cranial base and CPA procedures carry the risk of trigeminal nerve damage, and the incidence of de novo trigeminal symptoms following removal of cranial base meningiomas reaches 11% [109]. Consequences of intraoperative CN V damage include facial numbness, pain, decreased corneal sensitivity, disabled corneal reflexes, lacrimal dysfunction, hypersensitivity to light, cataracts, and corneal ulceration. Monitoring of CN V can be accomplished by using a free-running EMG or CMAP (trapezius muscle) following intracranial stimulation. Despite the ability to identify the nerve intraoperatively, the methods’ diagnostic value is questionable [110]. An alternative approach is monitoring the trigeminal SSEP [111, 112].

*Blink reflexes* are elicited by stimulation of the supraorbital branch of CN V, which induce a motor response in orbicular muscles consisting of a short-latency ipsilateral response (R1) of about 10 msec followed by bilateral polysynaptic responses of longer latency (R_2_~30 msec and R_3_~75–90 msec) [113]. The blink reflexes are extremely sensitive to anesthesia, however, application of stimulation trains allows for monitoring the R1 response in patients under TIVA or light inhalational anesthesia [113, 114].

Intraoperative monitoring of the blink reflex may become a valuable tool in neurosurgery, but the method needs validation in a larger group of patients.

### Monitoring CN VIII

**Figure 5.**
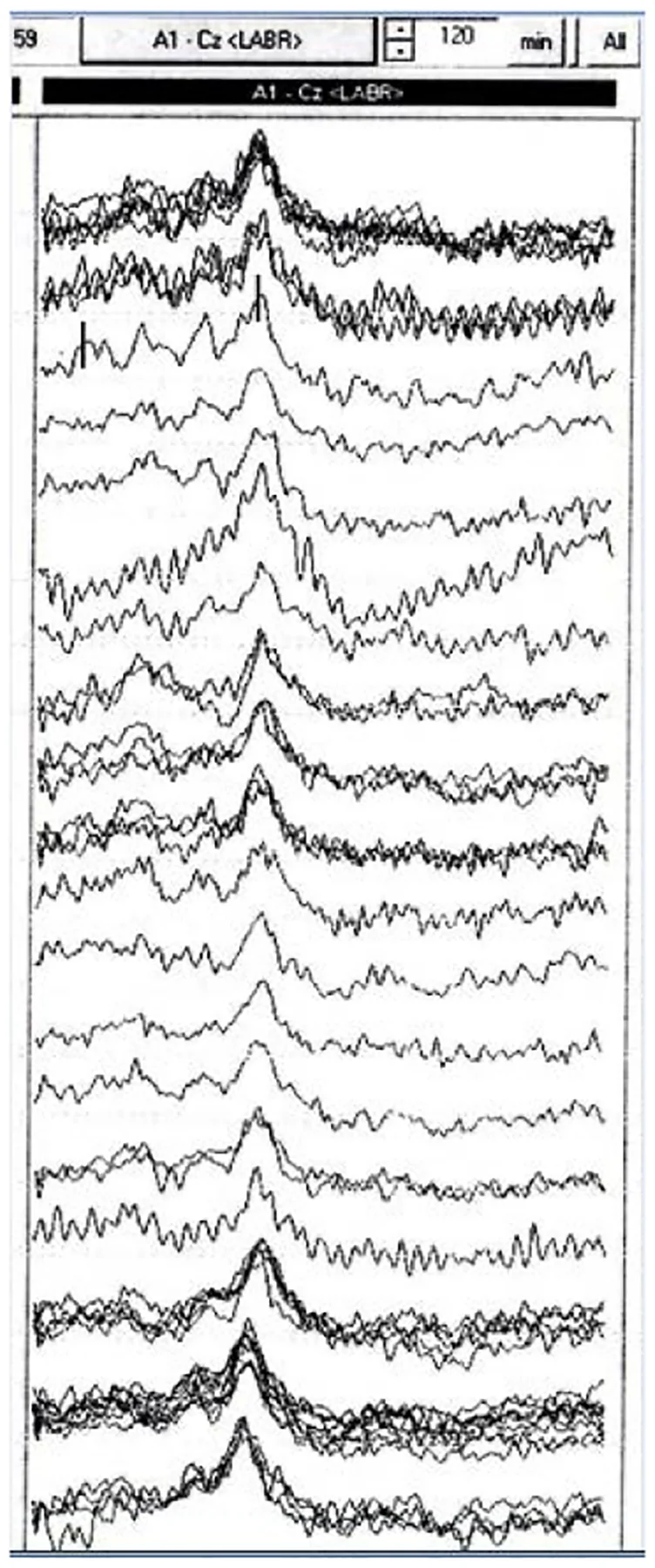
Stable brainstem acoustic evoked potentials (BAEP) in a patient undergoing surgery for hemifacial spasm (anesthesia: desflurane, remifentanil, propofol). Waveforms I and V are marked on figure from left to right.

BAEP are monitored during tumor removal at CPA, microvascular decompressions of CN VII and V (Figure 5), surgery on brainstem, and neurovascular interventions [5, 11, 97, 115–117]. BAEP are the least sensitive to changes in perioperative variables and drug actions [11]. However, conductive and sensorineural deafness on the surgery side preclude intraoperative BAEP monitoring.

The mechanisms of intraoperative acoustic nerve damage and hearing impairment are multiple and include cerebellar retraction with nerve stretching, direct mechanical trauma, electrical coagulation, high-energy electrical stimulation, and ischemia due to damage or vasospasm of the internal auditory artery [11, 26].

There are numerous studies showing improving hearing outcome with BAEP monitoring during acoustic neuroma surgery (as cited in [9, 11, 118]).

BAEP changes are assessed by comparing with the baseline recordings [16, 116]. Jahangiri et al. (2012) used the following criteria as warning signs during brainstem surgery: complete obliteration of peaks III and/or V and increase in peak V latency > 1 msec [95]. Others recommend a 50% decrease of peak amplitudes and >0.5 msec increase in wave V latency or even 20% changes in amplitudes and 0.1 msec delay in latency [16, 116].

IPL are less susceptible to host-related variables like age, gender and stimulation intensity when compared with peak V absolute latency [116]. However, the majority of these parameters are, at best, warning signs that alert the surgeon; among them only maintenance of peaks I and V has been consistently shown to correlate with better postoperative hearing preservation rates, although others have found poor correlation between postoperative hearing and BAEP [26].

False-positive and false-negative results of BAEP monitoring are explained by anesthesia, hypothermia, ear problems, as well as surgical manipulations not involving the auditory pathways and nuclei [16, 26, 115, 119, 118].

In contrast to BAEP, intraoperative ECoG and acoustic nerve CNAP record near-field potentials directly from the nerve, which ensures higher quality of signal and significant decrease in acquisition time [26]. While ECoG record the cochlear potential via transtympanic electrode, the CNAP are monitored directly from the acoustic nerve. In both cases, standard click stimulation is applied to generate the electrical potential.

Thus, BAEP, ECoG and CNAP are valuable modalities which can help to preserve postoperative hearing and prevent irreversible damage to brainstem structures.

### Monitoring CN III, IV and VI

The oculomotor, trochlear and abducens nerves are predominantly motor nerves with a few proprioceptive afferents terminating in the mesencephalic nucleus of CN V [120]. Intraoperative damage to these nerves will cause diplopia and seriously affect the lifestyle [120]. Both free-running EMG and CMAP after intracranial stimulation are used to monitor the oculomotor function and find a safe entry zone to the tegmental lesions [98, 121].

Electrooculography (EOG) is based on registration of the corneo-retinal electrical potential projected on the tissues surrounding the eye [120, 122, 123]. Eye movements cause orientation changes of the corneo-retinal potential thus allowing for monitoring spontaneous eye movements during surgery. In contrast to EMG, the method is noninvasive, and surface plate electrodes can be used to record the vertical and horizontal eye movements on separate channels. High amplitude (> 300 mcV) waves and trains of waves correlate with intraoperative tissue damage in the midbrain region, while small amplitude waves (50–150 mcV) and trains indicate possible trauma to the ponto-medullary area. EOG is not diagnostic for CPA pathology due to artifacts induced by irritation of the facial nerve [123].

A limitation of EOG is susceptibility to artifacts generated by electrical coagulation and stimulation.

## SSEP and MEP monitoring during brain surgery

SSEP are sensitive to changes in cerebral blood flow (CBF), although signal changes appear more slowly compared with EEG. [6, 9, 118]. In contrast to SSEP, EEG does not require averaging, covers larger cortical areas and is more sensitive to ischemia. However, SSEP is more specific and can detect ischemia in deeper structures including the brainstem. Unilateral SSEP changes on the side of surgery allow for differentiating between local ischemia caused by arterial clamping and global CBF derangement [124]. Combination of SSEP with MEP reduces the incidence of false-negative responses during carotid endarterectomy and helps to determine indications for temporary shunting [125, 126].

On the other hand, co-registration of SSEP with BAEP is 86% sensitive and 98% specific in predicting the neurological deficit during resection of cerebral AVM [115].

Surgery on cerebral aneurysms carries the risk of intraoperative ischemia in 5% of cases. (as cited in [19]) SSEP changes correlate with intraoperative CBF reduction, which makes that modality a valuable monitoring method during aneurysm surgery.According to Kang et al. (2013), SSEP monitoring is indicated when the aneurysms are supplied by internal carotid, anterior, middle, and posterior cerebral arteries [19]. Simultaneous BAEP recording may be required during posterior circulation surgery, even though-this combination yields an unacceptably high false-negative rate of 25% during basilar artery surgery (as cited in [127]). Accidental clamping of small perforator arteries during basilar aneurysm surgery may result in infarctions involving the thalamus, internal capsule, and midbrain, with resultant alterations in consciousness, hemiparesis or hemiplegia in the postoperative period. In such patients, combination of SSEP with TcMEP will improve the safety and increase the efficacy of INM [128].

Surgery in the territory of anterior cerebral arteries places at risk cortical areas controlling the lower extremities. In these cases, lower extremity SSEP is indicated, [9] while median nerve SSEP is the method of choice for aneurysms supplied by the middle cerebral and carotid arteries.

Significant SSEP changes during aneurysm surgery occur in 6.5% of cases [127]. Irreversible changes carry an 80% risk of postoperative stroke in patients with unruptured aneurysms, while the risk is 20% with reversible SSEP changes [127].

Intraoperative pneumocephalus, especially during sitting craniotomies, may significantly impact the SSEP registration [16, 129]. Multimodal neuromonitoring, which includes SSEP, direct electrical stimulation and MEP, has become an essential component during surgery in highly eloquent areas including cortical zones of language and motor control as well as deep-seated gliomas [3, 5, 95, 130–138].

During such interventions, ischemic events are the major source of permanent postoperative motor dysfunction, whereas, parenchymal resection represents a minor reason for neurological consequences [139]. A new motor deficit may be reversible if MEP recovery is achieved by early signs and appropriate modifications are made in the surgical procedure [110]. SSEP and MEP monitor distinct neuronal pathways, and both methods may be considered supplementary.

## Conclusion

Evoked responses are a valuable mode of INM. The selection of specific EP depends on location of the lesion, preexisting pathology, clinical experience with the method, and other factors. Sensitivity of the signal to general anesthetics and changes in physiological parameters during surgery must be taken into account. Advancements in INM technologies, neurosurgical technique and anesthesia significantly improve the quality and efficacy of neurosurgical procedures, reduce morbidity and improve the neurological outcome. Several above described methods still are not commonly used, and further research is required before their widespread application in clinical practice.

An active interaction between anesthesiologists, neurosurgeons and the neurophysiology team working in the operating room is a prerequisite for successful intraoperative EP monitoring and timely interpretation of the data.

## Abbreviations:

AEP: Auditory evoked potential(s)
AVM: Arterio-venous malformation
BAEP: Brainstem acoustic evoked potential(s)
CBF: Cerebral blood flow
CMAP: Compound muscle action potential(s)
CN: Cranial nerve(s)
CNAP: Compound nerve action potential(s)
CNS: Central Nervous System
CPA: Cerebellopontine angle
EEG: Electroencephalography
EMG: Electromyography
EOG: Electrooculography
EP: Evoked Potentials
HFS: Hemifacial spasm
ICP: Intracranial pressure
INM: Intraoperative neuromonitoring
IPL: Interpeak latency
MAC: Minimal alveolar concentration
MEP: Motor evoked potential(s)
MLAEP: Mid-latency auditory evoked potential(s)
MR: magnetic resonance
MVD: Microvascular decompression
SEP: Sensory evoked potential(s)
SHA: Superior hypophyseal artery
SSEP: Somatosensory evoked potential(s)
TcMEP: Transcranial motor evoked potential(s)
VEP: Visual evoked potential(s)
TES: Transcranial electrical stimulation
TMS: Transcranial magnetic stimulation
